# Supplementary material for: The phased pan-genome of tetraploid European potato
Source: Nature. 2025 Apr 16;642(8067):389–97. doi: 10.1038/s41586-025-08843-0 (PMC12158759; doi:10.1038/s41586-025-08843-0)
Supplement: Supplementary file 5 — Supplementary Figs. 3–14, 20, 21, 23–27 and 34–44. [file 41586_2025_8843_MOESM5_ESM.zip › suppl_figure_27.pdf]

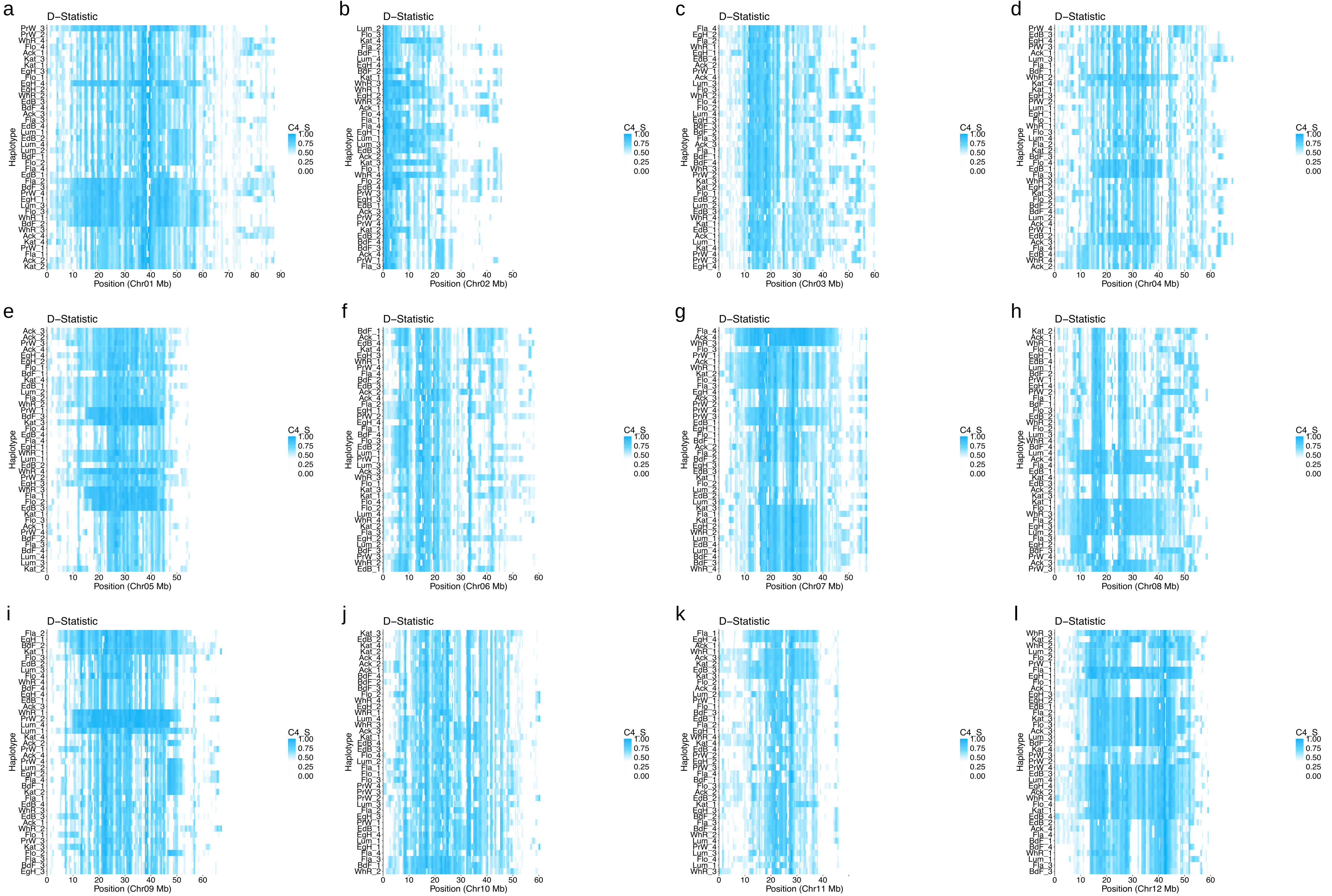
a
D–Statistic
Haplotype
Position (Chr01 Mb)
b
D–Statistic
Haplotype
Position (Chr02 Mb)
c
D–Statistic
Haplotype
Position (Chr03 Mb)
d
D–Statistic
Haplotype
Position (Chr04 Mb)
e
D–Statistic
Haplotype
Position (Chr05 Mb)
f
D–Statistic
Haplotype
Position (Chr06 Mb)
g
D–Statistic
Haplotype
Position (Chr07 Mb)
h
D–Statistic
Haplotype
Position (Chr08 Mb)
i
D–Statistic
Haplotype
Position (Chr09 Mb)
j
D–Statistic
Haplotype
Position (Chr10 Mb)
k
D–Statistic
Haplotype
Position (Chr11 Mb)
l
D–Statistic
Haplotype
Position (Chr12 Mb)
C4_S
1.00
0.75
0.50
0.25
0.00
